# Intake of Dietary One-Carbon Metabolism-Related B Vitamins and the Risk of Esophageal Cancer: A Dose-Response Meta-Analysis

**DOI:** 10.3390/nu10070835

**Published:** 2018-06-27

**Authors:** Yuzhen Qiang, Qianwen Li, Yongjuan Xin, Xuexian Fang, Yongmei Tian, Jifei Ma, Jianyao Wang, Qingqing Wang, Ruochen Zhang, Junhao Wang, Fudi Wang

**Affiliations:** 1 Department of Nutrition, Precision Nutrition Innovation Center, School of Public Health, Zhengzhou University, Zhengzhou 450001, China; qiangyzh@163.com (Y.Q.); lqw9319@163.com (Q.L.); yjxinzzu@163.com (Y.X.); tymayer@163.com (Y.T.); mjf15188351024@163.com (J.M.); 18734895045@163.com (J.W.); qingqingw820417@126.com (Q.W.); zhangruochen111@163.com (R.Z.); kevinwang93@163.com (J.W.); 2 Institute of Nutrition and Food Safety, School of Public Health, Zhejiang University School of Medicine, Hangzhou 310058, China; xuexianfang@zju.edu.cn

**Keywords:** esophageal cancer, dietary intake, one-carbon metabolism, B vitamins, dose-response meta-analysis

## Abstract

Several B vitamins are essential in the one-carbon metabolism pathway, which is central to DNA methylation, synthesis, and repair. Moreover, an imbalance in this pathway has been linked to certain types of cancers. Here, we performed a meta-analysis in order to investigate the relationship between the intake of four dietary one-carbon metabolism-related B vitamins (B2, B6, folate, and B12) and the risk of esophageal cancer (EC). We searched PubMed, Web of Science, and Embase for relevant studies published through 1 March 2018. The odds ratio (OR) with 95% confidence interval (CI) for the highest versus the lowest level of each dietary B vitamin was then calculated. From 21 articles reporting 26 studies including 6404 EC cases and 504,550 controls, we found an inverse correlation between the consumption of vitamin B6 and folate and the risk of EC; this association was specific to the US, Europe, and Australia, but was not found in Asia. A dose-response analysis revealed that each 100 μg/day increase in folate intake reduced the risk of EC by 12%. Moreover, each 1 mg/day increase in vitamin B6 intake decreased the risk of EC by 16%. Surprisingly, we found that each 1 μg/day increase in vitamin B12 intake increased the risk of esophageal adenocarcinoma by 2%, particularly in the US and Europe, suggesting both geographic and histological differences. Together, our results suggest that an increased intake of one-carbon metabolism-related B vitamins may protect against EC, with the exception of vitamin B12, which should be consumed in moderation.

## 1. Introduction 

Esophageal cancer (EC) is the ninth most common form of cancer and the sixth leading cause of cancer-related deaths worldwide, with an estimated 442,000 new cases and 440,000 deaths in 2013 [1]. In addition, EC was estimated to cause a loss of 9.8 million disability-adjusted life years, which places a major burden on healthcare systems around the world [1]. Thus, new approaches to prevent or reduce the risk of EC are urgently needed. 

Epidemiological studies have identified many factors associated with an increased risk of EC, including gender, obesity, alcohol consumption, tobacco smoking, gastroesophageal reflux disease, *Helicobacter pylori* infection, N-nitroso compounds, and micronutrient deficiency, with dietary factors appearing to play a significant role [2,3]. Therefore, diet-based preventive strategies have attracted considerable attention. In particular, four specific B vitamins—B2 (riboflavin), B6 (pyridoxine), B9 (folate), and B12 (cobalamin)—have attracted attention. These four vitamins act in concert to regulate the one-carbon metabolism pathway, which helps maintain nucleotide synthesis and methylation [4]. Accordingly, an imbalance and/or deficiency in any of these critical nutrients can interfere with DNA replication, DNA repair, and/or the regulation of gene expression, each of which can lead to carcinogenesis [5].

In recent decades, several epidemiological studies investigated the association between the risk of EC and intake of these B vitamins, yielding inconsistent results [6,7,8]. Several evidence-based studies have also evaluated this association. For example, data from meta-analyses suggest that an increased consumption of folate may decrease the risk of EC, while increased total vitamin B6 levels may help prevent many types of cancers, including EC [9,10,11]. On the other hand, no meta-analysis has been performed to examine the association between dietary vitamin B2 and vitamin B12 intake and the risk of EC. Thus, to the best of our knowledge, no systemic analysis has been performed regarding the putative association between dietary one-carbon metabolism-related B vitamins and the risk of EC. Therefore, we conducted a systematic meta-analysis in order to investigate this association.

## 2. Materials and Methods 

This meta-analysis was designed, implemented, analyzed, and reported in accordance with the Meta-analysis of Observational Studies in Epidemiology (MOOSE) protocol [12].

### 2.1. Search Strategy and Selection Criteria

PubMed, Web of Science, and Embase were systematically searched by two investigators (authors Y.Q. and Q.L.) for articles published through 1 March 2018, using the following search terms: (“vitamin B” or “vitamin B2” or “riboflavin” or “vitamin B6” or “pyridoxine” or “vitamin B9” or “folate” or “folic acid” or “vitamin B12” or “cobalamin”) AND (“esophagus” OR “esophageal” or “oesophageal”) AND (“cancer” OR “tumor” OR “carcinoma” OR “neoplasm”). In addition, the references cited within each relevant article were searched for additional eligible publications. Unavailable data in some articles were obtained by e-mailing the corresponding author. Unpublished studies were not included. The only language restriction was for articles published in English, and only full-text journal articles of original studies were included.

Eligible studies had to satisfy the following criteria: (1) the exposure was the intake of dietary vitamin B2, vitamin B6, folate, and/or vitamin B12; (2) the study reported the risk of EC using an odds ratio (OR), hazard ratio (HR), or relative risk (RR), with the corresponding 95% confidence interval (95% CI) for the highest level versus the lowest level of each one-carbon B vitamin intake (or presented data that could be used to calculate the risk estimate); (3) the outcome was EC, esophageal adenocarcinoma (EAC), or esophageal squamous cell carcinoma (ESCC); (4) the original study was either a case-control study or a cohort study; and (5) for the dose-response analysis, the intake of each B vitamin in each category was either provided or could be calculated. If the same data were used in more than one publication, the most recent complete study was included in our analysis. If a study reported more than one OR, HR, or RR, the most adjusted ratio was used to calculate the pooled OR.

We excluded meta-analyses, reviews, case-reports, articles for which the full text was not available, studies involving non-human species, articles lacking essential data, and other non-relevant publications. 

### 2.2. Data Extraction and Quality Assessment 

The following data were extracted from each eligible article by two investigators (authors Y.Q. and Q.L.): The first author’s name, publication year, country, geographic location, histological type, study design (hospital-based case-control (HBCC),population-based case-control (PBCC), or cohort study), source of controls (for HBCC and PBCC studies), age, sample size (number of participants, controls, and cases), dietary assessment, intake comparison, subclasses of dietary B vitamins, and the corresponding OR, HR, or RR with 95% CI. We extracted the OR, HR, and RR values with the most adjustment.

These two investigators also independently assessed the quality of each study using the Newcastle-Ottawa Scale [13]; any discrepancies were resolved through group discussion with a third investigator (J.M.). This scale assigns a maximum of nine points to each study, with a score of 0–3, 4–6, and 7–9 indicating low, moderate, and high quality, respectively. Any inconsistencies were resolved by group discussion. 

### 2.3. Statistical Analysis

In this meta-analysis, because the incidence of EC is relatively rare in all populations, the RR and HR were deemed equivalent to OR, and the summary results are reported as an OR for simplicity [14]. Pooled estimates of each OR and 95% CI were computed to assess the association between intake of each dietary one-carbon metabolism-related B vitamin and EC risk by comparing the highest level of consumption versus the lowest level of consumption. Any results that were stratified by histological type were treated as two independent reports.

We evaluated heterogeneity using the Q test and *I*^2^ statistic, with *I*^2^ values of 25%, 50%, and 75% representing low, moderate, and high degrees of heterogeneity, respectively [15]. If *I*^2^ > 50%, a DerSimonian and Laird random-effects model was used; otherwise, a Mantel-Haenszel fixed-effects model was used [16]. Meta-regression was performed in order to examine which possible sources of heterogeneity might have exerted a substantial impact on any between-study variation [17]. Subgroup analyses were also performed to evaluate the effect of modifying potential key covariates, stratified by geographic location (Asia, the US, Europe, or Australia), histological type (EAC or ESCC), study design (HBCC, PBCC, or cohort), dietary assessment (validated method, non-validated method, or not available [N/A]), and adjustment by energy intake/body mass index/alcohol/smoking (yes or no). 

We performed dose-response analyses for the risk of EC and the increased intake of vitamin B2, vitamin B6, folate, or vitamin B12 using the method recommended by Greenland and Longnecker and the publicly available Stata code written by Orsini et al. [18,19]. We extracted the range or mean intake of these B vitamins in each category, the number of cases and participants (or person-years) in each category, and the OR (or RR) with 95% CI. If the person-years/participant number in each category was not reported, groups were assumed to be of equal sizes [20]. If the data were not compared using the highest with lowest levels, an algorithm processor was used to transform a reference group containing discrete correlated data [21]. If neither median nor mean values were reported, we used the midpoint of the range. If the highest category was open-ended, we considered the width of that category to be the same as the width of the adjacent category. If the lowest category was open-ended, the lowest boundary was set to zero [22]. We evaluated possible nonlinear associations between the intake of dietary B vitamins and the risk of EC using restricted cubic splines, with three knots at the 10th, 50th, and 90th percentiles of the distribution [23]. A *p*-value for nonlinearity was calculated by testing the null hypothesis that the coefficient of the second spline was equal to zero.

A “leave-one-out” sensitivity analysis was used to evaluate whether the results would have been affected significantly by removing one study at a time. Publication bias was assessed using Egger’s test [24]. All data were analyzed using the statistical software program Stata, version 11.0 (StatCorp, College Station, TX, USA) and *p* < 0.05 was considered statistically significant.

## 3. Results

### 3.1. Search Results, Study Characteristics, and Quality Assessment 

**Figure 1:**
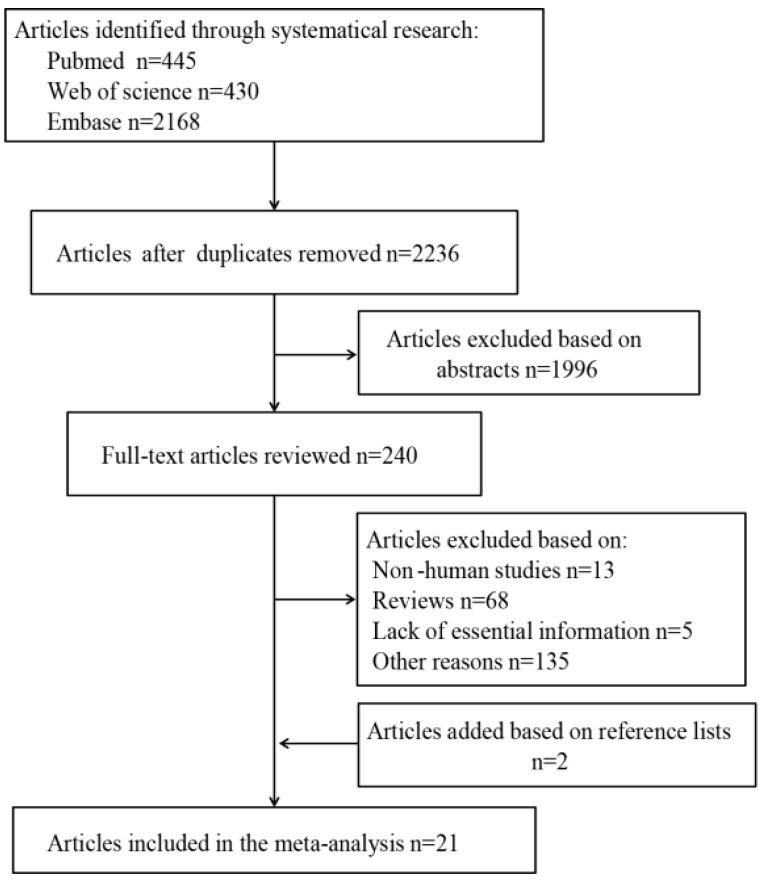
Flow diagram depicting the screening and exclusion of publications included in the meta-analysis.

The study selection process and the results of our literature search are shown in Figure 1. We initially identified 445 articles in PubMed, 430 articles in Web of Science, and 2168 articles in Embase. After excluding duplicates and studies that did not satisfy the inclusion/exclusion criteria, we identified 21 articles reporting 26 studies; 24 were case-control studies and two were cohort studies. 

Table 1 summarizes the characteristics of the 26 studies reported in the 21 articles included in our analysis. The studies included a total of 510,954 participants and 6404 cases, which included 1919 EAC patients, 2010 ESCC patients, and 2475 EC patients. For vitamin B2 intake, 14 studies with 3335 cases were included. Six of these studies were performed in the US [6,25,26,27,28], five in Europe [7,29,30,31], two in Australia [8] and one in Asia [32]. For vitamin B6 intake, 14 studies with 4151 cases were included. Seven of these studies were performed in the US [6,25,26,27,33], five in Europe [7,29,30,34,35], and two in Australia [8]. For folate intake, 21 studies with 5158 cases were included. Ten of these studies were performed in the US [6,25,26,27,28,33,36,37], six in Europe [7,29,34,38,39], three in Asia [40,41,42], and two in Australia [8]. For vitamin B12 intake, 10 studies with 3164 cases were included. Six of these studies were performed in the US [6,26,27,33], two in Europe [7,30], and two in Australia [8]. The quality scores of all 21 publications ranged from 6 to 8, with a median score of 7.

### 3.2. Dietary Vitamin B2 Intake and EC Risk

**Figure 2:**
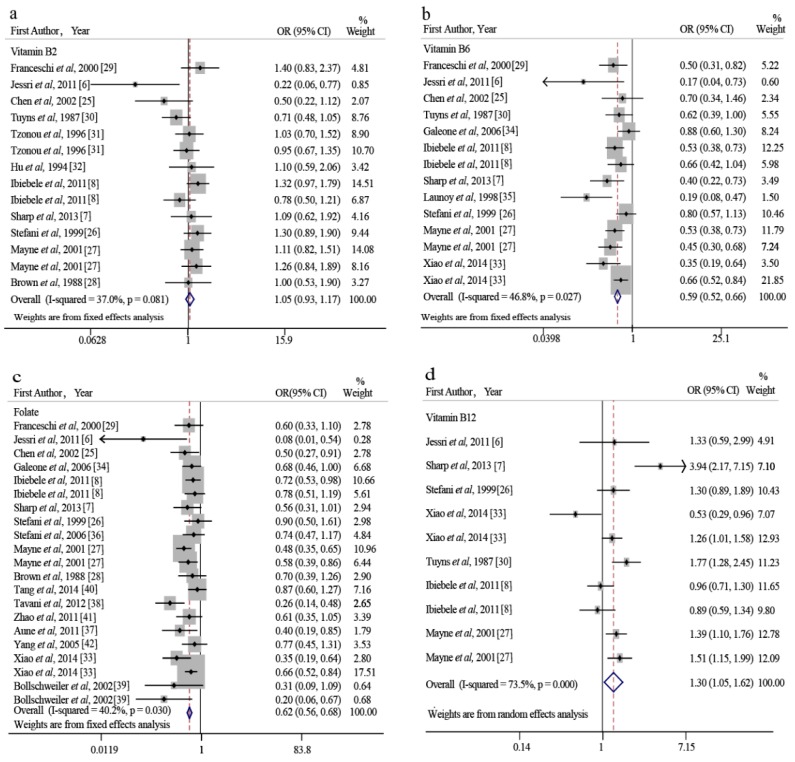
Forest plots summarizing the OR of EC for the highest vs. the lowest category of (**a**) vitamin B2 intake; (**b**) vitamin B6 intake; (**c**) folate intake; and (**d**) vitamin B12 intake. OR: odds ratio (relative risk); CI: confidence interval.

**Figure 3:**
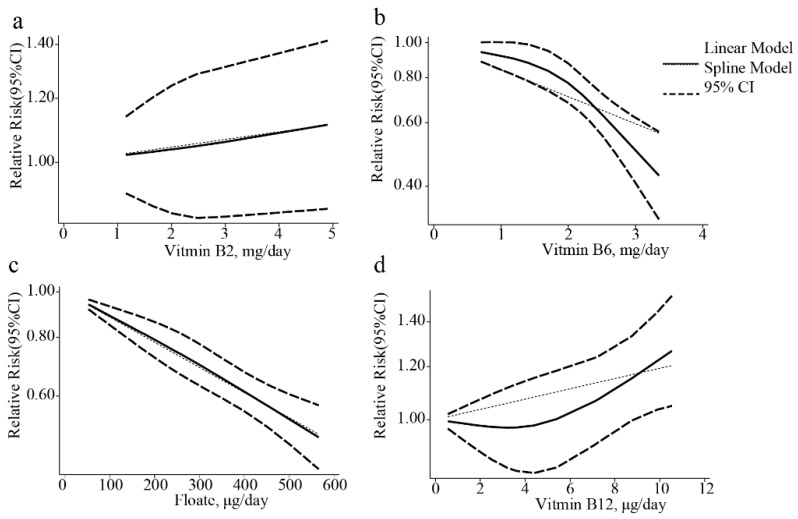
Dose-response relationships between EC risk and the daily intake of (**a**) vitamin B2; (**b**) vitamin B6; (**c**) folate; and (**d**) vitamin B12. In each panel, the solid line and dashed lines represent the estimated relative risk and 95% confidence interval, respectively, and the dotted line represents the linear fit to the data.

The pooled OR of EC risk for the highest level versus the lowest level of vitamin B2 intake was 1.05 (95% CI: 0.93–1.17; *I*^2^ = 37.0%) (Figure 2a). The results show that increasing vitamin B2 intake does not affect the risk of EC. A subgroup analysis revealed that only one study reported an inverse correlation between vitamin B2 intake and EC risk (OR: 0.22; 95% CI: 0.06–0.77); this study did not adjust for alcohol intake [6]. The results obtained using studies that did adjust for alcohol intake were similar to the overall results (OR: 1.06; 95% CI: 0.94–1.19; *I*^2^ = 18.1%; *p* = 0.261 versus the total pooled OR). Other results of our subgroup analyses were also consistent with the overall results (Table S1). A dose-response analysis of three studies revealed that each 1 mg/day increase in vitamin B2 intake had no effect on EC risk (OR: 1.01; 95% CI: 0.98–1.04). We also found no evidence of a nonlinear association between dietary vitamin B2 intake and EC risk (*p* = 0.932) (Figure 3a).

### 3.3. Dietary Vitamin B6 Intake and EC Risk

We found an inverse association between vitamin B6 intake and EC risk (OR: 0.59, 95% CI: 0.52–0.66; *I*^2^ = 46.8%) (Figure 2b). This inverse relationship remained significant when we performed subgroup analyses for EAC (OR: 0.58; 95% CI: 0.49–0.68) and ESCC (OR: 0.47; 95% CI: 0.33–0.67) (Table 2). Six studies were included in a dose-response analysis, which revealed a nonlinear relationship between dietary B6 intake and EC risk (*p* = 0.015; Figure 3b). The lowest level of dietary B6 intake (0.7 mg/day) was used as the reference dose in our dose-response analysis. When dietary vitamin B6 intake was ≥2.0 mg/day, the inverse association became significant. The OR (95% CI) of EC was 0.96 (0.97–1.00), 0.93 (0.88–0.99), 0.82 (0.76–0.88), 0.67(0.59–0.75), and 0.55 (0.44–0.67) for 1.2, 1.4, 2.0, 2.5, and 3.0 mg/day of dietary vitamin B6 intake, respectively (Figure 3b). This dose-response analysis indicates that each 1 mg/day increase in dietary B6 increase decreases the risk of EC by 16% (OR: 0.84; 95% CI: 0.80–0.89).

### 3.4. Dietary Folate Intake and EC Risk

We also found a significant inverse association between folate intake and EC risk, with a pooled OR of 0.62 (95% CI: 0.56–0.68; *I*^2^ = 40.2%) (Figure 2c). A subgroup analysis revealed that this inverse correlation was present in the US (OR: 0.58; 95% CI: 0.51–0.67), Europe (OR: 0.51; 95% CI: 0.40–0.65), and Australia (OR: 0.74; 95% CI: 0.58–0.95), but not in Asia (OR: 0.77; 95% CI: 0.59–1.01) (Table 2). A dose-response analysis including 13 studies revealed a linear relationship (*p* = 0.739) between dietary folate intake and the risk of EC. This dose-response analysis suggests that each 100 μg/day increase in folate intake reduces the risk of EC by 12% (OR: 0.88; 95% CI: 0.86–0.91) (Figure 3c).

### 3.5. Dietary Vitamin B12 Intake and EC Risk

Finally, we found a positive correlation between B12 intake and the risk of EC (OR: 1.30; 95% CI: 1.05–1.62; *I*^2^ = 73.5%) (Figure 2d). A subgroup analysis based on geographic location revealed similar results in the US (OR: 1.26; 95% CI: 1.03–1.53) and Europe (OR: 2.54; 95% CI: 1.16–5.53), but not in Australia (OR: 0.93; 95% CI: 0.73–1.19). A subgroup analysis based on histological type revealed that this correlation was present among patients with EAC (OR: 1.47; 95% CI: 1.02–2.11), but not among patients with ESCC (OR: 1.00; 95% CI: 0.63–1.61). A positive correlation was also found in the subgroup of studies that used a validated FFQ/DHQ (OR: 1.45; 95% CI: 1.10–1.91), but not in the subgroup of studies that did not use a validated FFQ/DHQ (OR: 1.03; 95% CI: 0.84–1.26). In addition, the significant association was only found in studies that adjusted for alcohol, smoking, daily energy intake, or BMI, but not in the other subgroup (Table S2).

A total of five studies were eligible for inclusion in a dose-response analysis, which revealed a positive linear association between dietary B12 intake and EC risk (*p* = 0.192). This dose-response analysis suggests that each 1 μg/day increase in dietary B12 intake increases the risk of EC by 2% (OR = 1.02; 95% CI: 1.00–1.03).

### 3.6. Heterogeneity and Meta-Regression

For the intake of three of the four dietary B vitamins, heterogeneity was <50%; in contrast, heterogeneity for vitamin B12 intake was 73.5% (*p* < 0.001) (Figure 2d). However, the source of heterogeneity between vitamin B12 intake and the risk of EC was not identified in a meta-regression analysis. Next, we performed subgroup analyses based on geographic location and histological type, revealing a positive association in the US and Europe but not in Australia, as well as in patients with EAC but not in patients with ESCC. Therefore, we conclude that both geographic location and histological type likely account—at least in part—for this relatively high heterogeneity. Moreover, a “leave-one-out” analysis revealed that the key contributors to heterogeneity were two studies by Sharp et al. and Xiao et al., which included extreme values without adjusting for smoking [7,33]. After excluding each of these studies, heterogeneity was reduced to 42.0%, and the summary OR for EC was 1.30 (95% CI: 1.17–1.45), which is consistent with the main finding.

### 3.7. Sensitivity Analysis and Publication Bias

A sensitivity analysis revealed that no individual study affected the pooled effect size (Figure S1 in Supplementary Materials). We then looked for publication bias using funnel plots (Figure S2) and Egger’s test. Based on Egger’s test, the *p*-value was <0.5 for vitamin B2, vitamin B6, and folate, but >0.05 for vitamin B12. We then excluded the study by Jessri et al., which did not adjust for alcohol, to determine whether this study was a source of bias [6] and found no significant publication bias in the final analysis with respect to vitamin B2 (*p* = 0.244), vitamin B6 (*p* = 0.068), folate (*p* = 0.054), or vitamin B12 (*p* = 0.093).

## 4. Discussion

This systemic meta-analysis was based on 26 studies including 510,954 participants and 6,404 cases of EC, revealing a clear correlation between the dietary intake of several one-carbon metabolism-related B vitamins and the risk of EC. Specifically, we found an inverse association between EC risk and vitamin B6 intake and folate intake specifically in the US, Europe, and Australia, but not in Asia. Moreover, our dose-response analysis revealed that each 1 mg/day increase in B6 intake and each 100 μg/day increase in folate intake reduces the risk of EC by 16% and 12%, respectively. To our surprise, however, we found that each 1 μg/day increase in B12 intake was associated with a 2% increase in the risk of EC, particularly in the US and Europe and particularly among patients with esophageal adenocarcinoma, suggesting both geography-specific and histology-specific effects. On the other hand, we found no significant association between dietary vitamin B2 intake and EC risk.

Vitamins B2, B6, B9 (folate), and B12 are essential nutrients involved in the one-carbon metabolism pathway, which plays a critical role in several key biological processes, including DNA stability and gene transcription [43], protein localization [44], and the degradation of small molecules [45]; thus, these nutrients are believed to play an important role in preventing cancer. The US Institute of Medicine’s Food and Nutrition Board established the following recommended daily allowance (RDA) for adults with respect to B vitamins: 1.3 and 1.1 mg/day of vitamin B2 for men and women, respectively; 1.3 mg/day of vitamin B6; 400 μg/day of folate; and 2.4 μg/day of vitamin B12 [46] . Moreover, observational studies reported that suboptimal levels of these B vitamins are associated with various types of cancers, including breast, colorectal, and lung cancer [47,48].

**Figure 4:**
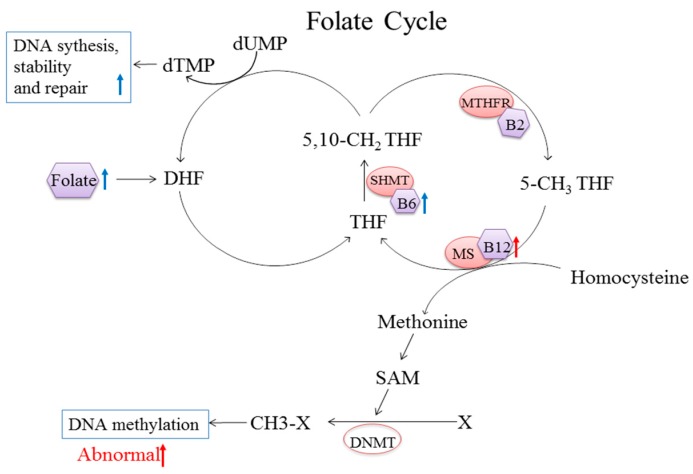
Schematic model of the folate-mediated one-carbon metabolism pathway and its relationship with methylation reactions. Folate undergoes two reduction steps to produce THF. Vitamin B6 serves as a co-factor of SHMT to catalyze the synthesis of 5,10-CH_2_ THF. Vitamin B2 serves as a precursor of the co-factor for MTHFR to produce 5-CH_3_ THF. The vitamin B12-dependant enzyme MS uses 5-CH_3_ THF to convert homocysteine to methionine, the precursor of SAM. Finally, SAM mediates various methylation reactions. The blue arrows indicate that high intake of vitamin B6 and folate leads to increased DNA synthesis, stability, and repair. The red arrows indicate that high intake of B12 is associated with abnormal DNA methylation. Abbreviations: DHF, dihydrofolate; THF, tetrahydrofolate; 5-CH_3_ THF, 5-methytetrahydrofolate; 5,10-CH_2_ THF, 5,10-methylene tetrahydrofolate; MTHFR, methylene tetrahydrofolate reductase; SHMT, serine hydroxymethyltransferase; MS, methionine synthase; SAM, S-adenosylmethionine; DNMT, DNA methyltransferase.

Several mechanisms might account for this relationship between the intake of these B vitamins and the development of cancer, as summarized in Figure 4. First, folate is the substrate for converting dUMP to dTMP, which is required for thymine synthesis and the formation and stability of DNA, RNA, and nucleoside triphosphates. Thus, folate deficiency may lead to the incorporation of uracil instead of thymine into the DNA and may alter DNA repair mechanisms, leading to chromosomal breakage [49]. Second, folate is a methyl donor, and vitamins B2, B6, and B12 serve as important co-factors for the enzymes methylene tetrahydrofolate reductase (MTHFR), serine hydroxymethyltransferase (SHMT), and methionine synthase (MS), respectively, in the folate cycle [50,51]. In addition, the vitamin B12-dependent enzyme MS catalyzes the conversion of homocysteine to methionine, which is required for synthesizing the universal methyl donor *S*-adenosyl methionine (SAM) and for the cellular circulation of folate [4,52]. Thus, a deficiency in B vitamins in the folate cycle can lead to the altered expression of critical proto-oncogenes and tumor suppressor genes by reducing DNA methylation, ultimately leading to the development of cancer [53]. However, it is worth noting that high intake of vitamin B12 may also cause higher levels of SAM, which could result in the increased activity of DNA methyltransferase (DNMT) enzymes [54]. Interestingly, DNMT1 plays a role in the self-renewal of cancer stem cells, which are involved in both tumorigenesis and tumor metastasis [55,56]. In addition, hypermethylation of the enzyme O^6^-methylguanine-DNA methyltransferase (MGMT) also has been correlated with mutations in the tumor suppressor protein p53 [57]. Thus, although the biology of these one-carbon metabolism-related B vitamins suggests that they confer a beneficial effect with respect to helping prevent cancer, vitamin B12 may paradoxically serve as a cancer-promoting factor.

In contrast with the effects of vitamin B6, folate, and vitamin B12 on EC risk, our analysis suggests that vitamin B2 (riboflavin) intake is not significantly associated with the risk of EC. Interestingly, previous studies have found that vitamin B2 deficiency was generally more severe in high-risk EC populations than in low-risk populations, which suggests that riboflavin deficiency may indeed play an important role in the etiology of EC [58]. However, an intervention study conducted in China found no significant difference in the incidence of ESCC between the intervention group and the control group after six years of consuming riboflavin-fortified salt [59]. Therefore, additional studies are needed in order to clarify the putative association between vitamin B2 intake and EC risk.

With respect to vitamin B6, our analysis suggests that adults should consume at least 2.01 mg/day in order to reduce the risk of EC. Nevertheless, it is important to note that this inverse relationship was observed in the US, Europe, and Australia, and no studies have yet been conducted in Asia; therefore, this result may not necessarily apply to Asian populations.

We also observed a significant inverse correlation between folate intake and EC risk, finding that each 100 μg/day increase in folate intake reduces the risk of EC by 12%. This finding is consistent with the results of a recent meta-analysis by Zhao et al. [11]. In the subgroup analysis, there was a geographic-specific difference which suggested that geographic location and/or dietary habits may play an important role in this association [9]. For example, people living in high-risk regions such as Asia may consume less folate-rich foods (e.g., green leafy vegetables, some fruits, legumes, and liver) compared to people living in low-risk regions [60]. Nevertheless, our analysis cannot take into consideration persons of Asian descent living in the US. Thus, additional studies are needed in order to determinate whether ethnicity plays a role.

Lastly, we found that vitamin B12 intake is positively associated with EC risk in both the US and Europe, but not in Australia. Based on the mean dose and number of participates in each category of vitamin B12 intake in the studies included, we estimated the average intake of dietary vitamin B12 in different geographical locations. We found that the value in Australia (2.17 μg/day) was much lower than that in the US (4.64 μg/day) and Europe (8.11 μg/day), which were both higher than the RDA of vitamin B12 for adults (2.4 μg/day) [46]. Interestingly, the positive correlation between vitamin B12 and EC risk was specific to esophageal adenocarcinoma, which has been increasing rapidly in prevalence in Western countries [2]. Vitamin B12 has been shown to regulate inflammation. For example, a recent cohort study found that vitamin B12 levels in the cerebrospinal fluid are positively correlated with 8-hydroxy-2-deoxyguanosine(8-OHdG), which is a marker of oxidative processes and is often related to inflammation [61]. Other studies found that vitamin B12 may play a role in regulating the pro-inflammatory cytokines IL-6 and TNF-α [62,63]. In addition, inflammation has been associated with an increased risk of EAC, leading to the proposed model of carcinogenic progression from inflammation (reflux esophagitis), to metaplasia (Barrett’s esophagus), to adenocarcinoma [64]. Moreover, when reflux esophagitis progresses to Barrett’s esophagus, the inflammatory response is skewed towards a more pronounced humoral immune response [65]. In this respect, it is interesting to note that vitamin B12 may serve as a modulator of the immune response. Tamura et al. found that vitamin B12 is related to CD8+ cells and NK cells [66], and Sukocheva et al. reported that vitamin B12 can regulate Ca^2+^ spikes in immune cells over a wide range of concentrations, possibly giving rise to physiological changes [67]. Thus, the role of vitamin B12 in inflammation and immune function may serve as an indirect link to EAC. Consistent with our results, an epidemiological intervention study found that supplementation with vitamin B12 was related to the risk of lung cancer [48]. In addition, the results of a randomized, placebo-controlled trial suggest that excess intake of vitamin B12 is associated with changes in DNA methylation in several genes that function during development, thereby reactivating and/or or deregulating their expression during carcinogenesis [68]. Considering the source of vitamin B12 from foods, meat and dairy products derived from foods of animal origins [60] are the main source. However, the fortified cereals are also an important source of dietary vitamin B12 [69,70]. Since we couldn’t identify the detailed information about the exact source of vitamin B12, we could not rule out the possibility of potential interaction between vitamin B12 and some ingredients in animal foods which are known risk factors for EAC [71]. Therefore, further well-designed studies are needed to address whether the food source is a confounding factor in the relationship between vitamin B12 intake and the risk of EC.

Our meta-analysis has several strengths. First, this is the first systemic meta-analysis that investigates the association between the intake of four one-carbon metabolic B vitamins and EC risk. Second, we performed several subgroup analyses and found significant geography-specific and histology-specific differences in the effects of both folate intake and vitamin B12 intake. Third, we performed the first dose-response meta-analysis of the quantitative effects of vitamins B2, B6, and B12 on EC risk.

Despite these strengths, our analysis has several limitations that warrant discussion. First, because most of the eligible studies were case-control studies, we are unable to rule out any possible effects of recall bias. In addition, the highest and lowest levels of vitamin B intake varied among the included studies; however, we do not believe that this affected our analysis, given the strength of our subgroup analysis.

## 5. Conclusions

In summary, the results of our meta-analysis indicate that both vitamin B6 intake and folate intake are inversely correlated with EC risk, whereas vitamin B12 intake is directly associated with EC risk; in contrast, we found no correlation between vitamin B2 intake and the risk of EC. In addition, our dose-response analyses support these general findings. Our results strongly suggest that increasing one’s daily dietary intake of vitamin B6 and folate help to reduce the risk of esophageal cancer. In contrast, higher intake of dietary vitamin B12 may increase the risk for esophageal cancer. Thus, large prospective cohort studies and randomized controlled trials are warranted in order to support these results and identify the underlying biological mechanisms.

## Figures and Tables

**Table 1:** Characteristics of the 21 articles included in the meta-analysis.

Author, Year	Country	Histological Type	Study-Design	Age Range (Years)	Participants (Cases)	Dietary Assessment	Subclass(es) of Vitamin B	Intake Comparison (Highest vs. Lowest)	Adjustment for Covariates	Quality Score
Franceschi et al., 2000 [29]	Italy	ESCC	HBCC	<79	1047 (304)	FFQ-78 items, validated	Vitamin B2	Q5 vs. Q1	Age, gender, area of residence, education, physical activity, BMI, tobacco smoking, alcohol drinking and non-alcohol energy.	7
							Vitamin B6			
							Folate			
Jessri et al., 2011 [6]	Iran	ESCC	HBCC	40–75	143 (47)	FFQ-125 items, validated	Vitamin B2	T3 vs. T1	Age (years), sex (male/female), gastroesophageal reflux disease symptoms, BMI, smoking status, smoking intensity, smoking duration, physical activity, and education level.	8
							Vitamin B6			
							Folate			
							Vitamin B12			
Chen et al., 2002 [25]	US	EAC	PBCC	>21	573 (124)	DHQ, validated	Vitamin B2	Q4 vs. Q1	Age, age squared, gender, respondent type, BMI, alcohol use, tobacco use, education level, family history of respective cancers, and vitamin supplement use.	7
							Vitamin B6			
							Folate			
Tuyns et al., 1987 [30]	France	Mix-type	PBCC	N/A	2718 (743)	DHQ-40 items, validated	Vitamin B2	>2.0 mg/day vs. <1.5 mg/day	Age, alcohol consumption, and tobacco smoking.	6
							Vitamin B6	>3.0 mg/day vs. <2.0 mg/day		
							Vitamin B12	>10.0 μg/day vs. <5.0 μg/day		
Tzonou et al., 1996 [31]	Greece	ESCC/EAC	HBCC	N/A	243 (43)/256 (56)	FFQ-115 items, validated	Vitamin B2	Q5 vs. Q1	Gender, age, birthplace, schooling, height, analgesic use, coffee drinking, alcohol intake, tobacco smoking, and energy intake (though not mutually analyzed).	6
Hu et al., 1994 [32]	China	Mix-type	HBCC	N/A	588 (196)	FFQ-32 items, N/A	Vitamin B2	Q4 vs. Q1	Alcohol intake, smoking, household income, and occupation.	7
Launoy et al., 1998 [35]	France	ESCC	HBCC	<85	607 (208)	DHQ-39 items, N/A	Vitamin B6	>2.5 mg/day vs. <1.5 mg/day	Interviewer, age, smoking, beer intake, aniseed aperitifs, hot Calvados, whisky, total alcohol intake, and total energy intake.	7
Galeone et al., 2006 [34]	Italy and Swiss	ESCC	HBCC	<80	1226 (351)	FFQ-78 items, validated	Vitamin B6	>2.249 mg/day vs. <1.722 mg/day	Education, BMI, tobacco smoking, and alcohol drinking.	8
							Folate	>305.1 μg/day vs. <228.1 μg/day		
Ibiebele et al., 2011 [8]	Australia	ESCC/EAC	PBCC	18–79	1732 (225)/2120 (613)	FFQ-135 items, N/A	Vitamin B2	4.9 mg/d vs. 1.65 mg/day	Age, gender, education, BMI 1 year previously, frequency of heartburn or acid reflux 10 year prior to diagnosis, lifetime alcohol intake, pack-years of smoking, NSAID use, and total energy intake.	7
							Vitamin B6	2.3 mg/day vs. 0.7 mg/day		
							Folate	504.5 μg/day vs. 136.0 μg/day		
							Vitamin B12	4.95 μg/day vs. 0.55 μg/day		
Sharp et al., 2013 [7]	Ireland	EAC	PBCC	≤85	479 (223)	FFQ-101 items, validated	Vitamin B2	≥2.8 mg/day vs. ≤1.8 mg/day	Age, sex, total energy, years of full-time education, BMI, and alcohol intake.	8
							Vitamin B6	≥3.2 mg/day vs. ≤2.3 mg/day		
							Folate	≥421 μg/day vs. ≤318 μg/day		
							Vitamin B12	≥9.7 μg/day vs. ≤6.4 μg/day		
Stefani et al., 2006 [36]	Uruguay	ESCC	HBCC	N/A	1266 (234)	FFQ-64 items, validated	Folate	Q4 vs. Q1	Age, sex, residence, urban/rural status, birthplace, education, BMI, smoking status, years since quitting smoking, number of cigarettes smoked per day, alcohol drinking, *mate* consumption, and total energy intake.	7
Mayne et al., 2001 [27]	US	ESCC/EAC	PBCC	30–79	893 (206)/969 (282)	FFQ-104 items, validated	Vitamin B2	75th vs. 25th	Sex, location, age, race, proxy status, income, education, usual BMI, cigarettes smoked/day, years of consuming beer, wine, and/or hard liquor, and energy intake.	8
							Vitamin B6			
							Folate			
							Vitamin B12			
Tang et al., 2014 [40]	China	Mix-type	HBCC	N/A	739 (359)	FFQ-137 items, validated	Folate	>204.5 μg/day vs. <104.5 μg/day	Age, gender, education level, BMI, total energy intake, smoking status, alcohol drinking, and family history of cancer in first-degree relatives.	6
Tavani et al., 2012 [38]	Italy	Mix-type	HBCC	N/A	1767 (505)	FFQ-78 items, validated	Folate	≥312.5 μg/day vs. ≤257.3 μg/day	Sex, age, study center, year of interview, education, alcohol drinking, tobacco smoking, BMI, total energy intake, and physical activity at work.	7
Zhao et al., 2011 [41]	China	ESCC	HBCC	37–75	465 (155)	FFQ-45 items, N/A	Folate	>300 μg/day vs. <230 μg/day	Age, sex, smoking, and drinking.	7
Brown et al., 1988 [28]	US	Mix-type	PBCC	≤79	629 (207)	DHQ, N/A	Vitamin B2	high vs. low	Use of cigarettes and/or alcohol.	6
							Folate			
Aune et al., 2011 [37]	Uruguay	Mix-type	HBCC	<90	2266 (234)	FFQ-64 items, N/A	Folate	275.31 μg/day vs. 123.83 μg/day	Age, sex, residence, education, income, interviewer, smoking status, cigarettes per day, duration of smoking, age at starting smoking, years since quitting smoking, calcium, dietary fiber, and iron intake, *mate* drinking, BMI, and energy intake.	7
Stefani et al., 1999 [26]	Uruguay	Mix-Type	HBCC	N/A	459 (66)	FFQ-64 items, N/A	Vitamin B2	T3 vs. T1	Age, sex, residence, urban/rural status, education, BMI, tobacco smoking (in pack-years), alcohol drinking, and total energy intake.	6
							Vitamin B6			
							Folate			
							Vitamin B12			
Yang et al., 2005 [42]	Japan	Mix-type	HBCC	18–80	660 (165)	FFQ-47 items, validated	Folate	>400 μg/day vs. <300 μg/day	Smoking, drinking, and total energy,	6
Bollschweiler et al., 2002 [39]	Germany	ESCC/EAC	PBCC	>40	102 (52)/97 (47)	FFQ-110 items, validated	Folate	>164 μg/day vs. 0–120 μg/day	N/A	6
Xiao et al., 2014 [33]	US	ESCC/EAC	Cohort	50–71	490,780 (185)/491,169 (574)	FFQ-124 items, validated	Vitamin B6	2.7 mg/day vs. 1.4 mg/day	N/A	7
							Folate	566 μg/day vs. 288 μg/day		
							Vitamin B12	7.3 μg/day vs. 2.5 μg/day		

Abbreviations: BMI, body mass index; EAC, esophageal adenocarcinoma; ESCC, esophageal squamous cell cancer; PBCC, population-based case-control; HBCC, hospital-based case-control; DHQ, Dietary History Questionnaire; FFQ, Food Frequency Questionnaire; NSAID, nonsteroidal anti-inflammatory drug; N/A, not available.

**Table 2:** Subgroup analyses between the intake of three dietary one-carbon metabolism-related B vitamins and the risk of EC.

	Vitamin B6						Folate						Vitamin B12				
	N	Cases/controls	OR (95%CI)	*I* ^2^	*p*-Value		N	Cases/Controls	OR (95%CI)	*I* ^2^	*p*-Value		N	Cases/Controls	OR (95%CI)	*I* ^2^	*p*-Value
Overall	14	4151/497,974	0.59 (0.52–0.66)	46.8%	0.027		21	5158/501,583	0.62 (0.56–0.68)	40.2%	0.030		10	3164/495,508	1.30 (1.05–1.62)	73.5%	0.000
**Geographic Location**																	
Asia	-	-	-	-	-		3	679/1185	0.77 (0.59–1.01)	0.0%	0.575		-	-	-	-	-
America	7	1484/492,219	0.59 (0.51–0.69)	49.1%	0.067		10	2159/496,392	0.58 (0.51–0.67)	37.3%	0.110		6	1360/492,457	1.26 (1.03–1.53)	50.7%	0.071
Europe	5	1829/4253	0.51 (0.34–0.78)	66.6%	0.017		6	1482/3186	0.51 (0.40–0.65)	49.8%	0.076		2	966/2231	2.54 (1.16–5.53)	81.2%	0.021
Australia	2	838/1507	0.57 (0.44–0.74)	0.0%	0.442		2	838/1507	0.74 (0.58–0.95)	0.0%	0.764		2	838/1507	0.93 (0.73–1.19)	0.0%	0.771
**Histological Type**																	
EAC	5	1816/493–493	0.58 (0.49–0.68)	0.0%	0.487		6	1863/493,544	0.60 (0.51–0.69)	34.1%	0.181		4	1692/493,245	1.47 (1.02–2.11)	82.9%	0.001
ESCC	7	1526/494–901	0.47 (0.33–0.67)	64.7%	0.009		9	1759/495,894	0.61 (0.51–0.73)	28.2%	0.194		4	663/492,884	1.00 (0.63–1.61)	74.55%	0.008
**Dietary Assessment (FFQ/DHQ)**																	
Validated	10	3039/495–675	0.58 (0.50–0.66)	39.4%	0.095		15	3658/497,556	0.59 (0.52–0.66)	49.1%	0.017		7	2260/493,608	1.45 (1.10–1.91)	75.8%	0.000
N/A	4	1112/2299	0.56 (0.38–0.83)	68.2%	0.024		6	1500/4664	0.70 (0.58–0.85)	0.0%	0.641		3	3164/1900	1.03 (0.84–1.26)	8.4%	0.335
**Study Design**																	
HBCC	4	910/2113	0.41 (0.20–0.84)	77.2%	0.004		9	2354/7225	0.59 (0.44–0.77)	55.3%	0.022		1	47/380	1.33 (0.59–2.99)	-	-
PBCC	8	2482/5267	0.58 (0.50–0.67)	5.8%	0.385		10	2045/3764	0.61 (0.52–0.70)	22.8%	0.233		7	2358/5505	1.42 (1.10–1.84)	75.6%	0.000
Cohort	2	758/490,594	0.51 (0.28–0.94)	73.0%	0.054		2	759/490,594	0.51 (0.28–0.94)	73.0%	0.054		2	759/490,594	0.86 (0.37–1.99)	85.5%	0.008
**Samples**																	
≥500	11	3815/497–229	0.58 (0.51–0.65)	40.9%	0.076		15	4568/500,478	0.62 (0.42–0.76)	38.7%	0.063		7	2828/494,763	1.19 (0.96–1.47)	70.5%	0.002
<500	3	336/745	0.47 (0.23–0.98)	72.1%	0.028		6	590/1105	0.48 (0.30–0.78)	51.4%	0.068		3	336/745	1.89 (0.90–3.98)	80.0%	0.007

Abbreviations: EAC, esophageal adenocarcinoma; ESCC, esophageal squamous cell cancer; PBCC, population-based case-control; HBCC, hospital-based case-control; DHQ, Dietary History Questionnaire; FFQ, Food Frequency Questionnaire; N/A, not available; OR, odds ratio; CI, confidence interval.

## Supplementary Materials

The following are available online at http://www.mdpi.com/2072-6643/10/7/835/s1: Figure S1: Sensitivity analysis in which one study was removed at a time in order to evaluate the stability of the results. (a) Vitamin B2 intake; (b) vitamin B6 intake; (c) folate intake; and (d) vitamin B12 intake, Figure S2: Funnel plots for the intake of B vitamins and the risk of esophageal cancer. (a) Vitamin B2 intake; (b) B6 intake; (c) folate intake; and (d) vitamin B12 intake, Table S1: Subgroup analysis of the intake of vitamin B2 and the risk of EC, Table S2: Subgroup analysis of the intake of B vitamins and the risk of EC.
