# Supplementary material for: Intake of Dietary One-Carbon Metabolism-Related B Vitamins and the Risk of Esophageal Cancer: A Dose-Response Meta-Analysis
Source: Nutrients. 2018 Jun 27;10(7):835. doi: 10.3390/nu10070835 (PMC6073467; doi:10.3390/nu10070835)
Supplement: Supplementary file 1 [file nutrients-10-00835-s001.zip › EES Certificate Qiang et al.pdf]

ENGLISH EDITING SOLUTIONS

English Editing Solutions has edited the manuscript originally entitled "Dietary one-carbon metabolism-related B vitamins intake and the risk of esophageal cancer: A dose-response meta-analysis" by Qiang et al. The main text has been edited for grammar, spelling, punctuation, and sentence structure. This certificate is not an endorsement of the manuscript's contents, and the conclusions and opinions expressed in the manuscript are the sole opinion and property of its authors.

June 16, 2018

Curtis F. Barrett, PhD
English Editing Solutions
www.EnglishEditingSolutions.com
