# Supplementary material for: Intake of Dietary One-Carbon Metabolism-Related B Vitamins and the Risk of Esophageal Cancer: A Dose-Response Meta-Analysis
Source: Nutrients. 2018 Jun 27;10(7):835. doi: 10.3390/nu10070835 (PMC6073467; doi:10.3390/nu10070835)
Supplement: Supplementary file 1 [file nutrients-10-00835-s001.zip › TableS1.pdf]

TableS1. Subgroup analysis of dietary one-carbon metabolism-related vitamin B2 intake and the risk of EC.

Subgroups	N	Cases/controls	OR(95%CI)	I^2	*P*
**Overall**	**14**	**3335/7120**	**1.05(0.93,1.17)**	**37.0%**	**0.081**
**Geographic Locations**					
Asia	1	196/392	1.10(0.59,2.06)	-	-
America	6	932/2047	0.98(0.72,1.35)	55.6%	0.046
Europe	5	1369/3174	0.97(0.80,1.17)	12.3%	0.335
Australia	2	838/1507	1.04(0.62,1.74)	72.9%	0.055
**Dietary Assessment**					
Validated FFQ/DHQ	9	2028/4406	0.99(0.85,1.15)	46.0%	0.063
N/A FFQ/DHQ	5	1307/2714	1.15(0.95,1.39)	8.3%	0.359
**Histologic Type**					
EAC	5	1298/3099	1.09(0.92,1.29)	30.1%	0.221
ESCC	5	825/3233	0.98(0.69,1.39)	58.3%	0.048
**Study Design**					
HBCC	6	712/1824	1.08(0.89,1.30)	41.9%	0.126
PBCC	8	2623/5296	1.03(0.89,1.19)	41.0%	0.105
**Sample**					
≥500	9	2900/6175	1.05(0.91,1.21)	39.0%	0.108
**Adjusted dietary energy intake**					
Yes	8	1714/3043	1.11(0.98,1.27)	0.0%	0.583
no	6	1621/4077	0.82(0.55,1.20)	55.6%	0.046
**Score**					
≥7	7	1382/2623	1.08(0.90,1.31)	46.5%	0.082
<7	7	1953/4453	1.02(0.88,1.19)	34.9%	0.162
**Adjusted BMI**					
Yes	9	2090/4131	1.12(0.97,1.29)	48.9%	0.048
No	5	1245/2989	0.92(0.78,1.12)	0.0%	0.659
**Adjusted alcohol**					
Yes	13	2188/7024	1.06(0.94,1.19)	18.1%	0.261
no	1	47/96	0.22(0.06,0.77)	N/A	N/A
**Adjusted smoking**					
Yes	13	3112/6864	1.04(0.93,1.17)	41.8%	0.056
no	1	233/256	1.09(0.62,1.92)	N/A	N/A
<500	5	435/945	1.04(0.85,1.27)	46.8%	0.111

Abbreviations: EC, esophageal cancer; EAC, esophageal adenocarcinoma; ESCC, esophageal squamous cell cancer; PBCC, population-based case-control; HBCC, hospital-based case-control; F/M, female and male; M, male; DHQ, Dietary History Questionnaire; FFQ, Food Frequency Questionnaire; N/A, not available.
