# Supplementary material for: Intake of Dietary One-Carbon Metabolism-Related B Vitamins and the Risk of Esophageal Cancer: A Dose-Response Meta-Analysis
Source: Nutrients. 2018 Jun 27;10(7):835. doi: 10.3390/nu10070835 (PMC6073467; doi:10.3390/nu10070835)
Supplement: Supplementary file 1 [file nutrients-10-00835-s001.zip › TableS2.pdf]

TableS2. Subgroup analysis of dietary one-carbon metabolism-related vitamins B intake and risk of esophageal cancer.

	Vitamin B6					Folate					Vitamin B12				
	N	Cases/ controls	OR(95%CI)	I^2	P	N	Cases/ controls	OR(95%CI)	I^2	P	N	Cases/ controls	OR(95%CI)	I^2	P
**Overall Score**	14	4151/497974	0.59(0.52,0.66)	46.8%	0.257	21	5158/501583	0.62(0.56,0.68)	40.2%	0.030	10	3164/495508	1.30(1.05,1.62)	73.5%	0.000
≥7	10	2504/494099	0.51(0.40,0.64)	55.4%	0.017	15	3523/498386	0.55(0.49,0.62)	36.7%	0.076	6	1517/491633	1.39(1.01,1.92)	78.1%	0.000
＜7	4	1647/3875	0.64(0.53,0.78)	0.0%	0.398	6	1635/3197	0.78(0.65,0.93)	0.0%	0.966	4	1647/3875	1.19(0.87,1.63)	69.2%	0.021
**Adjusted BMI**															
Yes	10	2441/5006	0.59(0.52,0.68)	39.1%	0.097	14	3773/9712	0.62(0.55,0.70)	46.8%	0.027	7	1662/3626	1.37(1.05,1.79)	72.5%	0.001
No	4	1710/492968	0.47(0.30,0.73)	68.8%	0.022	7	1385/491871	0.61(0.51,0.73)	33.4%	0.173	3	1502/492569	1.13(0.69,1.87)	83.5%	0.002
**Adjusted alcohol**															
Yes	11	3345/497229	0.58(0.51,0.67)	44.0%	0.057	16	4253/10843	0.63(0.57,0.71)	27.0%	0.149	7	2358/4818	1.42(1.10,1.84)	75.6%	0.000
no	3	336/745	0.43(0.22,0.83)	69.6%	0.037	5	905/490740	0.35(0.19.0.65)	66.1%	0.019	3	806/490690	0.97(0.55,1.73)	72.2%	0.028
**Adjusted smoking**															
Yes	11	3169/7124	0.59(0.52,0.67)	47.6%	0.039	16	2413/10683	0.63(0.56,0.71)	40.0%	0.050	7	2182/4658	1.31(1.16,1.48)	49.9%	0.063
no	3	982/490850	0.49(0.31,0.76)	62.4%	0.070	5	1081/490900	0.57(0.47,0.70)	48.4%	0.101	3	982/490850	1.38(0.56,3.36)	90.9%	0.000
**Adjusted dietary energy intake**															
Yes	7	1823/3242	0.58(050,0.66)	39.4%	0.095	11	3112/8044	0.63(0.56,0.72)	49.4%	0.032	6	1615/2843	1.37(1.03,1.83)	77.1%	0.001
no	7	2328/494732	0.56(0.38,0.83)	68.2%	0.024	10	2046/493539	0.59(0.50,0.69)	31.8%	0.154	4	1549/492665	1.17(0.77,1.79)	75.3%	0.007
**Sample**															
≥500	11	3815/497229	0.58(0.51,0.65)	40.9%	0.076	15	4568/500478	0.62(0.42,0.76)	38.7%	0.063	7	2828/494763	1.19(0.96,1.47)	70.5%	0.002
<500	3	336/745	0.47(0.23,0.98)	72.1%	0.028	6	590/1105	0.48(0.30,0.78)	51.4%	0.068	3	336/745	1.89(0.90,3.98)	80.0%	0.007

Abbreviations: OR, odds ratio; CI, confidence interval.
