# Supplementary figures and images for: Intake of Dietary One-Carbon Metabolism-Related B Vitamins and the Risk of Esophageal Cancer: A Dose-Response Meta-Analysis
Source: Nutrients. 2018 Jun 27;10(7):835. doi: 10.3390/nu10070835 (PMC6073467; doi:10.3390/nu10070835)

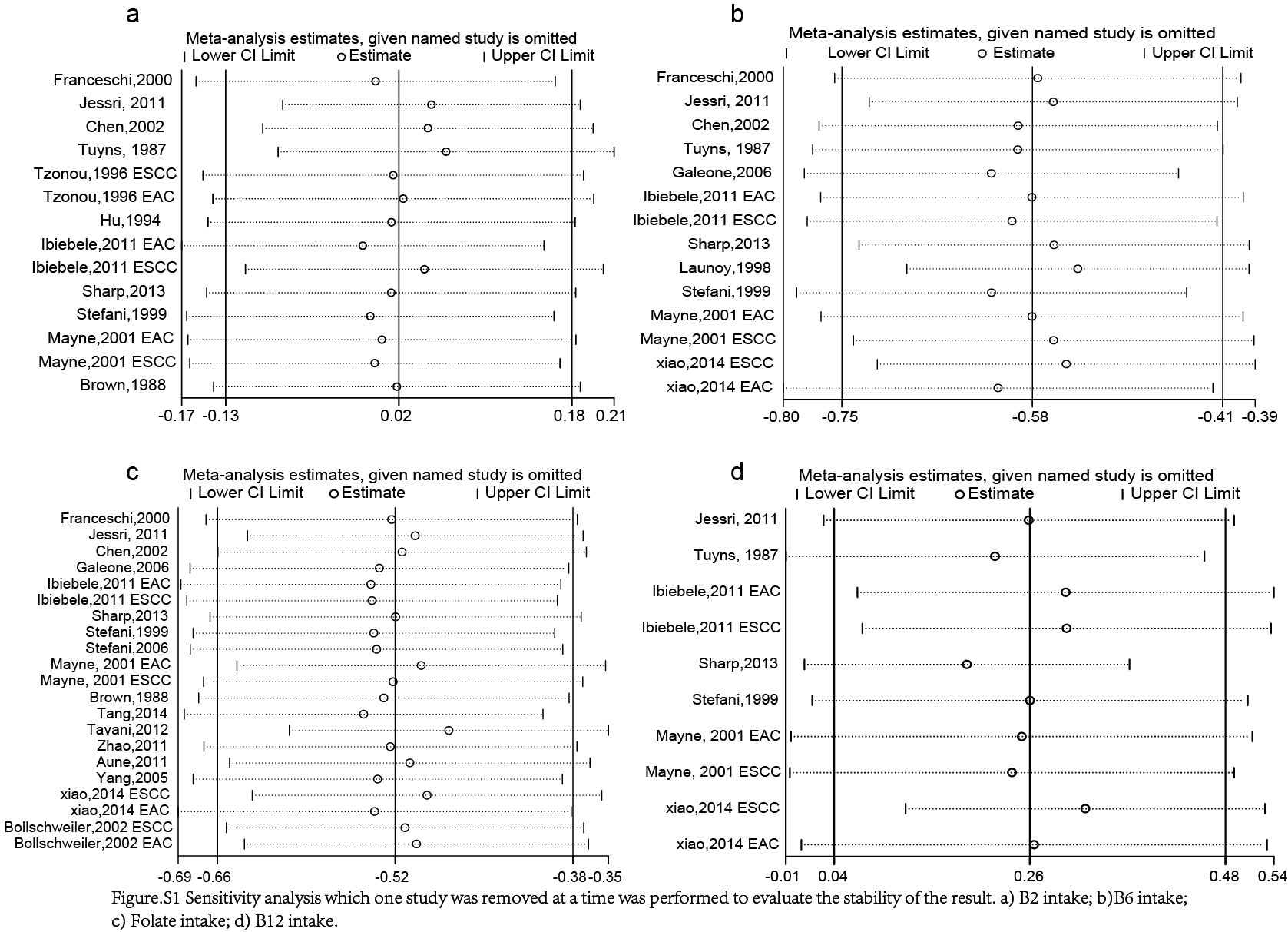

Supplement: Supplementary file 1 [file nutrients-10-00835-s001.zip › figureS1.png]

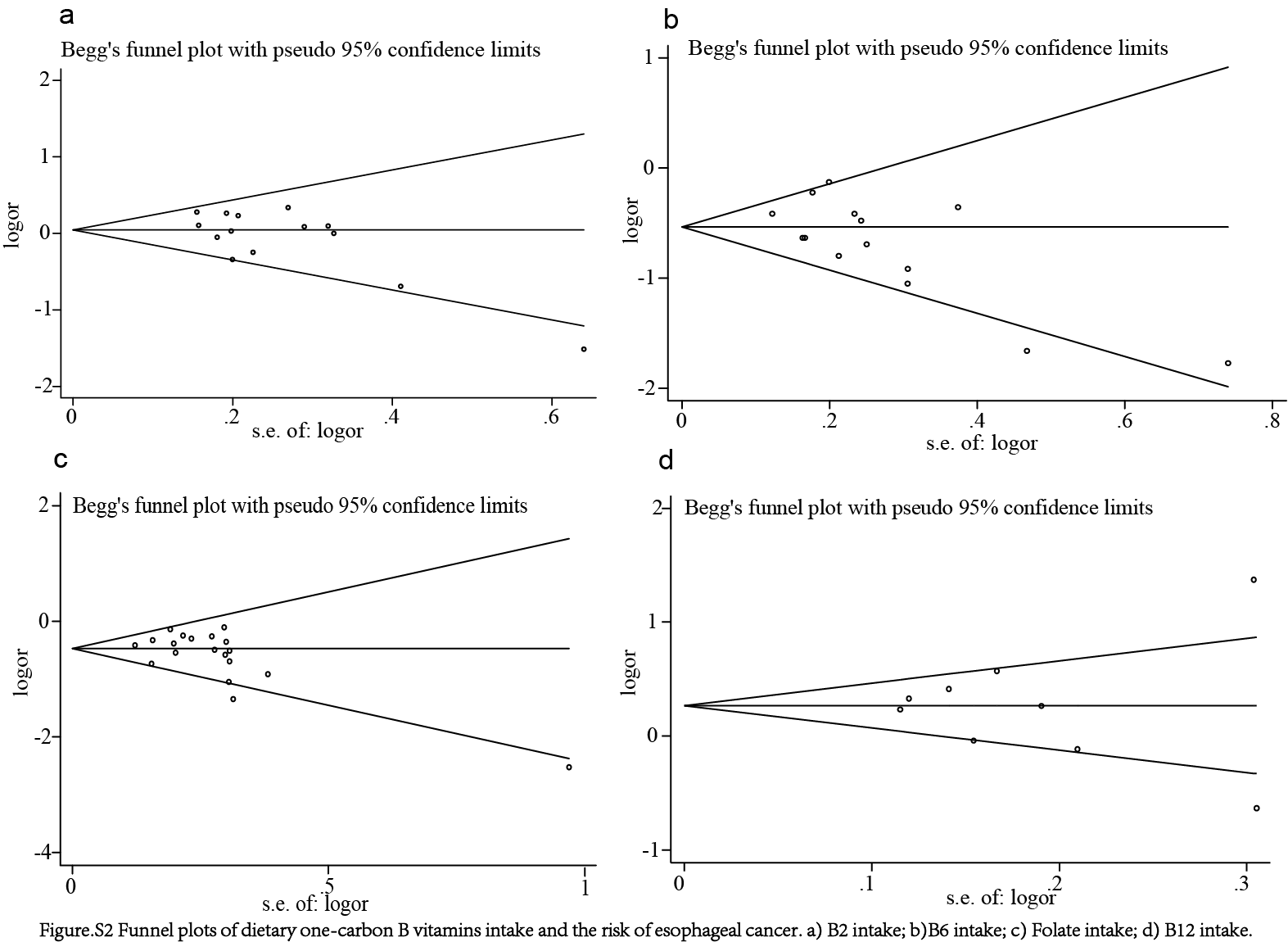

Supplement: Supplementary file 1 [file nutrients-10-00835-s001.zip › figureS2.png]
